# The Need to maintain Intraocular Pressure over 24 Hours

**DOI:** 10.5005/jp-journals-10008-1118

**Published:** 2012-10-16

**Authors:** Shibal Bhartiya, Parul Ichhpujani

**Affiliations:** Glaucoma Facility, Department of Ophthalmology, Fortis Memorial Research Institute, Gurgaon, Haryana, India; Glaucoma Facility, Department of Ophthalmology, Government, Medical College and Hospital, Chandigarh, India

**Keywords:** Diurnal variation, Fluctuation, Intraocular pressure, Phasing.

## Abstract

Glaucoma patients who appear to be stable based on daytime in-clinic intraocular pressure (IOP) measurements may not be fully controlled over each 24-hour period. Given that there is sufficient evidence that IOP fluctuation may impact progression, the aim of management of glaucoma thus, is to achieve a target IOP with minimal diurnal fluctuation.

**How to cite this article:** Bhartiya S, Ichhpujani P. The Need to Maintain Intraocular Pressure over 24 Hours. J Current Glau Prac 2012;6(3):120-123.

## INTRODUCTION

Intraocular pressure (IOP) is the most important and the only modifiable, known risk factor for glaucoma^1-8^ and consequently, most therapeutic interventions are directed at its modification.^1-31^ IOP is regulated by three factors: The rate of aqueous humor formation, the resistance to aqueous humor outflow and the episcleral venous pressure.

Before we proceed further, let’s understand the gamut of terminology pertaining to the IOP variables:

*IOP peak*: The highest IOP recorded in a stated time period.

*IOP trough*: The lowest IOP recorded in a stated time period.

*Circadian fluctuation*: The range of IOP during a 24-hour time period and is a specific type of short-term IOP fluctuation.

*Nocturnal fluctuation or nocturnal variation*: The range of IOP during typical sleeping hours within a 24-hour time period and is a specific type of short-term IOP fluctuation.

*Short-term IOP fluctuation*: IOP peak minus IOP trough, measured in a stated time period, within a 24-hour time period (intravisit).

*Long-term IOP fluctuation*: IOP peak minus IOP trough measured in a stated time period, on separate days (intervisit).

The IOP variables, such as peak levels^2-4,18,21^ and fluctuations (both short-term and over longer periods)^15,19,23^ have been known to adversely impact the disease development and progression, even in cases with ‘statistically’ normal or ‘controlled’ pressures.^1,2,6,9,10,15,20,30^

Most glaucomatologists concur that IOP peaks tend to be associated with visual field decline,^10,22,31^ but there is little consensus as regards IOP fluctuation as a risk factor for progression of glaucoma.^10,11,13-16,19,23,26^

This article attempts to elucidate the role of round the clock IOP control and its relevance to current glaucoma practice, as well as emerging therapeutic and diagnostic techniques.

*Scientific evidence so far:* The efficacy of IOP reduction in retarding the progression of glaucoma, over a wide spectrum of disease, from low to high IOPs, and from early to advanced disease has been conclusively documented in literature.^2-4,18,21^

*Advanced glaucoma intervention study (mean follow-up of 7.4 years)*: Long-term IOP fluctuation was associated with visual field progression in subjects with low mean IOP but not in patients with high mean IOP.^15^

*Early manifest glaucoma trial (median follow-up, 8 years), malmo ocular hypertension study (mean follow-up, 8.5 years), ocular hypertension treatment study*: Diurnal and long-term IOP fluctuations were not found to be significant risk factors for progression of early glaucoma or ocular hypertension.^12,13,23^

Lowering IOP by at least 18% (mean) from baseline has been shown to result in at least a 40% reduction in rates of worsening of glaucoma over 5 years.^2,5,18,21,28^

*Mean IOP*: Mean IOPs of those who progress to blindness, however, have been reported to not differ from those who do not, with the severity of glaucoma at the time of diagnosis and the range of IOPs found during follow-up (long-term IOP fluctuation) being important predictive factors.^17,27^

*Amount of IOP fluctuation*: Evaluation of patients treated surgically who achieved low IOPs found that the group with lower IOP fluctuation demonstrated significantly better preservation of the VF than the group with higher fluctuation, with similar mean IOP and number of glaucoma medications.^19,23^

*IOP peak*: IOP peaks during periods of noncompliance or loss of IOP control may therefore, indirectly be held responsible for glaucomatous progression in patients who otherwise have good IOP control.

### IOP Variation and Visual Field Progression

A strong correlation between fluctuation/variation and visual field progression has been reported by Asrani et al. However, the study was flawed in its design with assessment of IOP and visual field progression being cross-sectional and longitudinal, respectively. Also, IOP was measured using home tonometry, and the IOP fluctuation during a particular 5-day period may not be considered indicative of fluctuation/variation in that same patient over several years during which period visual field progression may or may not have occurred.

Most of the evidence regarding IOP and fluctuation and variation from large prospective, multicenter randomized clinical trials is posthoc analyses. Since, these studies did not necessarily address initial study questions and thus these results and conclusions may not be accorded the same status as analyses from the studies with the same as primary study goals.^1,2,4-7,13,15,18,21,24,32^

### How does an Increased Fluctuation of IOP Affect the Progression of Visual Field Loss?

Morgan et al have demonstrated that portions of the lamina cribrosa move maximally during pressure changes of 5 to 7 mm Hg, in contrast to minimal movement at pressure changes exceeding 15 mm Hg.^33^ Hence, there is a possibility that kinking of the axons may occur in small pockets of the lamina cribrosa, which move maximally at small pressure changes while other pockets may remain relatively stationary.

Another proposed theory is that IOP fluctuations may result in an ischemia reperfusion injury. Researchers provided evidence of this nature when they demonstrated that patients with higher fluctuations of IOP have damaged DNA in their circulating lymphocytes.^34^

Another significant pressure fluctuation involves the phenomenon of IOP spikes that occurs when people awake. Individuals with normal trabecular outflow typically experience a spike of approximately 6 mm Hg upon waking. This IOP rise dissipates in about 12 minutes, and the IOP returns to presleep levels. However, in a patient with glaucoma who has an impaired aqueous outflow mechanism, the spike of IOP may be higher and may take much longer to dissipate.

### Which is More Important: Peak IOP, Mean IOP or Fluctuating IOP?

Despite the fact that there is no conclusive evidence in literature to show the relation between fluctuation of IOP and glaucoma progression, there is considerable interest in the former. Since, the realative importance of the IOP attributes with regard to damaging the retinal ganglion cells and optic nerves is yet to be ascertained, the aim of management of glaucoma should target to favorably modify all three parameters: peak IOP, mean IOP and fluctuating IOP.^35^

### What is a ‘Practical’ DV Protocol for Clinical Practice?

Diurnal IOP assessment is costly, time-consuming and inconvenient for patients and may not be possible in most outpatient clinics. World Glaucoma Association guidelines advocate a minimum of IOP measurements at 08:00, 12:00, 16:00 and 20:00 to assess its diurnal variation.

An early morning recording of IOP, with the patient in supine position, has an additional advantage of detecting IOP spikes coincident with increased cortisol levels just before the patient wakes up. In clinics with inpatient facilities, the same may provide useful information.

### ‘Phased’ Diurnal Variation

There is fair to good agreement for IOP at any given time on different days, which makes a ‘phased’ diurnal variation of IOP a valid choice. Therefore, it is advisable to call the patient at different times, on successive days as a surrogate measure of diurnal variation of IOP.

This IOP measurement over several days may be a more realistic sampling of the patient’s IOP as the patients experience less modification in their normal routine.^38^ The patient’s compliance with medications and physiologic changes (e.g. hormonal or metabolic) that may take place over days and weeks rather than hours, especially since they strongly correlate to a diurnal curve, is also better assessed.

### Is One DV Enough?

There is evidence that diurnal IOP pattern from day to day is not repeatable in eyes of healthy individuals as well as treated primary open-angle glaucoma (POAG) patients, and that the assessment of single-day IOP variation poorly characterizes short-term IOP variation.^36-40^

There is a poor repeatability between the IOP dips and spikes on different days in both normal subjects, and glaucoma patients when using Goldmann applanation tonometry.^36,37^ In a small series of seven international airline pilots, Daubs evaluated diurnal IOP patterns using the Durham–Langham pneumatic-type electronic tonometer, and reported limited repeatability of IOP patterns.^39^ Twenty-four hour IOP data in normal eyes for three consecutive days using Schiotz tonometry also showed poor repro-ducibility of 24-hour IOP range.^40^

### How does the Management Protocol change based on Available Evidence?

The IOP should be measured at the same time of day both before and after initiating therapy to minimize inter-day variation, so that the changes observed after initiating therapy may be directly attributable to treatment efficacy.

The setting of IOP measurements (e.g. office, sleep lab, home), number and timing of measurements obtained are all important in assessment of fluctuations of IOP.

Since, the current data suggests that repeatability of IOP change overtime is uniformly poor, it is important to repeat diurnal IOP recordings in case a patient continues to deteriorate inspite adequate diurnal pressure control, and in all patients with advanced disease.

When resources do not permit 24 hours IOP recording, phased IOP records and/or office time IOP records may be considered as alternatives.

## CONCLUSION

IOP is not a static number; instead, it tends to fluctuate throughout the 24 hours. Mean IOP is a strong predictor of glaucomatous damage. A desired therapeutic target is therefore a uniform reduction of IOP throughout the 24 hours.
